# Intimate partner violence help-seeking attitudes in the Democratic Republic of the Congo: a population-based analysis

**DOI:** 10.1186/s12889-025-24148-3

**Published:** 2025-09-30

**Authors:** Shannon N. Wood, Anaise Williams, Pierre Z. Akilimali, Dynah Kayembe, Suzanne O. Bell, Michele R. Decker

**Affiliations:** 1 Department of Population, Family and Reproductive Health, Johns Hopkins Bloomberg School of Public Health, Baltimore, USA; 2 William H. Gates Sr. Institute for Population and Reproductive Health, Johns Hopkins Bloomberg School of Public Health, Baltimore, USA; 3 Kinshasa School of Public Health, Kinshasa, Democratic Republic of Congo; 4 Johns Hopkins School of Nursing, Baltimore, USA

**Keywords:** Gender-based violence, Intimate partner violence, Help-seeking, Attitudes, Democratic Republic of Congo

## Abstract

**Background:**

Intimate partner violence (IPV) is the leading form of gender-based violence globally, and is particularly pronounced in the Democratic Republic of the Congo (DRC), where 36% of women report experiencing physical and/or sexual IPV within the past 12 months. Despite increased attention to IPV service provision, help-seeking remains suboptimal. In DRC provinces of Kongo Central and Kinshasa, we examined: 1) characteristics of IPV and related help-seeking, 2) women’s attitudes towards IPV help-seeking, and 3) the association between IPV help-seeking attitudes and help-seeking.

**Methods:**

A population-based reproductive health survey conducted in Kongo Central and Kinshasa provinces of the DRC from December 2021-February 2022 examined past-year IPV and related help-seeking among partnered women participating in a gender-based violence module (*n* = 707 Kinshasa; *n* = 692 Kongo Central). Descriptive analyses examined forms of IPV (physical, sexual, emotional), help-seeking (informal/formal sources), and help-seeking attitudes. Among women who experienced IPV, bivariate and multivariable logistic regression examined individual help-seeking attitudes on help-seeking experience.

**Results:**

Approximately one in three women reported past-year IPV (35.3% in Kinshasa_,_ 29.7% in Kongo Central). Among past-year IPV survivors, 73.1% in Kinshasa and 62.3% in Kongo Central sought no help; for women who did seek help, less than 2% were from formal sources. Attitudes around women seeking help from police and women’s groups were largely negative (i.e., women did not feel these sources could help). Disagreeing or strongly disagreeing with attitude “a woman who seeks help from the police for domestic violence shames her family” was associated with increased odds of help-seeking in multivariable pooled models (aOR:1.73; 95% CI: 1.03, 2.89; *p* < 0.05).

**Conclusions:**

These findings reiterate the need to dismantle harmful gender systems and promote survivor-centered services. Confidence in services and corresponding attitudes favorable of seeking help are necessary to promote help-seeking for this leading form of gender-based violence.

## Background

Gender-based violence (GBV) is a leading cause of women’s morbidity and mortality globally [1, 2]. Intimate partner violence (IPV) is the most pervasive form of GBV—one in three women will experience IPV over the course of their lifetimes [2], and this abuse has detrimental implications for women’s sexual, reproductive, and mental health [3, 4].

Despite IPV’s commonality and health effects, care-seeking for IPV experiences is poorly understood globally [5]. Recent efforts have sought to expand IPV service provision and access, including medical, psychosocial, legal, and housing services [6–8]—some countries have gone as far as to consolidate IPV services into one-stop centres [9], or integrate IPV care within existing services to ease the accessibility burden for survivors [10]. Obtaining medical and/or psychosocial support can reduce revictimization [11–14] and mental health impacts, including post-traumatic stress [15] and self-blame [16], however, such services are rarely sought [5] and benefits are variable on type and quality of services [17]. In low- and middle-income contexts, in particular, approximately 3% of IPV survivors seek help from formal services, and instead most opt to turn first to trusted informal supports [18, 19], including family members [5] and friends [20].

Recent global studies have sought to better understand barriers to seeking help from formal services, including those related to shame and blame [21–24]. This research positions harmful gender norms as an underlying macro-level factor that can stigmatize women for the abuse they experience and inhibit them from seeking help due to their own normalization of abuse, self-blame, and/or fear of retaliation from their partner [25, 26]. Similarly, providers embedded within these harmful gender structures may exhibit attitudes that shame and blame IPV survivors for the abuse they experience [27]. Notably, while some providers do verbally or nonverbally express these attitudes during interactions with IPV survivors, survivors’ own perceptions of others’ attitudes (i.e., anticipated stigma) may be enough to prevent them from seeking support services in the first place [28]. While globally most norms surrounding IPV and its acceptance are rooted in underlying gender and power structures [29], context-specific research can help understand barriers to help-seeking, including harmful attitudes that may inhibit care-seeking, in settings where services are known and available to IPV survivors [21].

The Democratic Republic of the Congo (DRC) is an IPV hotspot, where recent World Health Organization data report that 36% of ever-partnered women aged 15–49 in the DRC experienced physical and/or sexual violence within the past 12 months—the highest past year prevalence of IPV globally [30]. Most recent Demographic and Health Survey data indicate that lifetime help-seeking for IPV survivors in the DRC was 38.5%, with 14.9% seeking help from a formal institution [5, 31]. Investment into violence prevention and response in the country has largely occurred in Eastern provinces given historical humanitarian crises, specifically multi-national wars and their sequelae that have plagued the country for over three decades [32]—this conflict has notably been linked to heightened IPV and non-partner sexual violence through perpetuation of harmful gender norms, including acceptance of violence [33–35]. Reports, however, indicate that IPV is pervasive country-wide [30, 36]. The Joint Program DRC Fight Against GBV strategy launched in 2018 and has focused on upgrading health facilities for medical and psychosocial care for GBV survivors, alongside strengthening legal response systems [37]. Despite investments, the national health system infrastructure remains under-resourced for adequate response, as exhibited by severe health worker shortages and large-scale financing via out-of-pocket payments and external aid [38]. Together, these data contribute to the DRC’s 151 st Gender Inequality Index ranking out of 179 countries [36], underscoring a critical need to increase gender equity, including by understanding drivers of violence, barriers to accessing care, and attitudes that may perpetuate reluctance to seek help. Notably, many formal sources of help are available within the DRC, specifically in health facilities [37]. Utilizing population-based data from two provinces of the DRC (Kinshasa and Kongo Central), the aims of this study were to understand: 1) the prevalence and characteristics of IPV and associated help-seeking; 2) women’s attitudes towards IPV help-seeking; and 3) among women who experienced IPV, how attitudes surrounding IPV are related to seeking help for their experiences.

## Methods

### Study design and sample

The present analyses use data from Performance Monitoring for Action (PMA), a research platform that administers annual panel and cross-sectional surveys in eight countries in sub-Saharan Africa and Asia. In the DRC, PMA uses a multi-stage cluster design with probability proportional to size sampling of enumeration areas to obtain regionally representative estimates of Kinshasa and Kongo Central provinces. Eligible study participants include females aged 15–49 within selected households who provided informed verbal/written consent. Surveys are conducted in-person by locally trained female enumerators. The female survey collected information on the respondents’ sociodemographic characteristics, reproductive history, family planning attitudes and use, and women’s empowerment. All study procedures and survey methodology for the PMA study is available at www.pmadata.org.

The data for this study come from Phase 3 of the DRC survey collected from December 2021-February 2022, where we included a module on GBV. For respondent safety, only one woman per household was eligible to complete the GBV module, selected randomly via Open Data Kit (ODK) software in cases of multiple eligible participants. IPV items were then only asked to women married or those currently living with a partner as if married, for a final analytic sample of *n* = 703 (99.4% response rate) in Kinshasa and *n* = 684 (98.8% response rate) in Kongo Central. As consistent with PMA procedures, all surveys were translated and back-translated. Prior to survey implementation, a two-week training emphasized best practices for research on violence against women, including non-judgmental survey implementation and referral to local support organizations. Referrals were offered to all women, regardless of violence disclosure and were included in a larger pamphlet on reproductive health and COVID-19 related services to not draw suspicion. Pre-testing of measures was done with resident enumerators as part of the training to ensure accuracy of item meaning within the local context.

### Measures

#### Attitudes towards IPV response

Five items on help-seeking attitudes from the IPV Help-Seeking Norms Scale [21] were selected based on local priorities: 1) A woman who seeks help from the police for domestic violence (DV) shames her family; 2) A person who intervenes when a woman is being beaten by her husband would be considered to be interfering in the couple’s private affairs; 3) Mediation is the best solution for families experiencing DV; 4) Women's groups that get involved in a DV case usually make the situation worse; and 5) Communities must help women get the help they need in situations of violence. Response options captured level of agreement with each statement (1-strongly agree, 2-agree, 3-neutral, 4-disagree, 5-strongly disagree). We constructed dichotomous versions of each item (1 = strongly agree, agree, neutral vs. 0 = disagree or strongly disagree).

#### Past-year IPV experience

The survey asked five items derived from the Revised Conflict and Tactics Scale (CTS-2) [39] on husband or partner behaviors in the last 12 months: 1) insulted her, yelled at her, screamed or made humiliating remarks; 2) slapped, hit or physically hurt her; 3) threatened with a weapon or attempted to strangle or kill her; 4) pressured or insisted on having sex when she did not want to (without physical force); or 5) physically forced her to have sex when she did not want to. We then created binary measures for any IPV (affirmative response to any of the 5 items), emotional IPV (affirmative response to item 1), physical IPV (affirmative response to items 2 or 3), sexual IPV (affirmative response to items 4 or 5), and contact IPV (affirmative response to any of the items 2 through 5); binary measures for types of violence (emotional, physical, sexual) were not mutually exclusive.

#### Past-year help-seeking for IPV

All women who indicated any past-year IPV experience were asked a single item on help-seeking: “Thinking about the experiences of relationship conflict we have just discussed, have you tried to seek help in the last 12 months?” For women who indicated past-year help-seeking, specific services sought were then probed upon; services were not mutually exclusive. We categorized sources of care into formal (doctor/medical professional, police, lawyer, social service organization, or a violence support program/hotline) and informal (own family, husband’s/partner’s family, current/former husband/partner/boyfriend, friend, neighbor, or religious leader). For each source of help sought, usefulness of the help was assessed via item: “How helpful was seeking help from this person/service?” with response range: 1-not helpful, 2-a little helpful, 3-neither helpful or not helpful, 4-helpful, or 5-very helpful. For visual depiction, usefulness was coded as 0 = not helpful, a little helpful, or neither helpful or not helpful vs. 1 = helpful or very helpful. For regression analyses, a binary dependent variable for any past-year help-seeking was constructed (“1” if the respondent answered “yes” to seeking any help and “0” if no help was sought).

#### Respondent characteristics

Respondent characteristics assessed as covariates utilized standard assessment [40, 41], and included household wealth (low, middle, high), marital status (married vs. living with partner), age [15–49], education (primary or lower vs. secondary or higher), parity (0, 1–2, 3 +), age at first intercourse (< 15, 15–17, > 17), whether she had paid work outside the household (yes/no), whether she had a savings account (yes/no), whether she uses a mobile money account (yes/no), partner education (secondary or lower vs. tertiary), whether her partner has other partners (yes/no), and age of first marriage (< 18 vs. >= 18).

### Statistical analysis

Analyses were conducted per province, when possible. First, descriptive analyses explored sample characteristics and prevalence of any IPV, any emotional IPV, any physical IPV, any sexual IPV, and any contact IPV (physical or sexual). Among those who experienced IPV, any help-seeking, type of help seeking (formal or informal), and the number of informal types of help accessed was reported. Among women who experienced IPV, the percent of women who reported useful help, per source of help sought, was visually depicted.

Exploratory factor analysis with promax factor rotation was then used to explore psychometrics of a potential scale for the five IPV response attitudes items. Given that the factor analysis suggested no underlying latent factor (highest eigenvalue: 0.65_Kinshasa_, 0.80_KongoCentral_), items were further explored independently, rather than as a scale. Among the full sample, participant agreement for each help-seeking attitude was visually displayed. Lastly, among women who experienced IPV, bivariate logistic regressions were run between each attitude item (independent variables—dichotomized as agree/neutral vs. disagree) and having sought help (dependent variable). Pooled multivariable models with a fixed effect for province were additionally explored, run separately for each attitude item (independent variable) and help-seeking (dependent variable), adjusting for key demographic variables.

All analyses were conducted using Stata 15.1 (College Station, TX) with statistical significance set a priori at p < 0.05. There were < 2% missing values for respondent characteristics variables in both settings; as such, complete case analysis was used and regression analyses float to accommodate small amounts of missing data. All estimates are weighted to account for the complex survey design.

## Results

Table 1 presents characteristics of partnered women interviewed for the GBV module, by province. Slightly more than half of the Kinshasa sample (56.2%) and slightly less than half of the Kongo Central sample (41.4%) were married to, as opposed to living with, their partner. Both samples were balanced across age groups, and the majority of women in both provinces were parous. Substantial provincial differences were observed in educational attainment—while 91.1% of women in Kinshasa had a secondary or higher education, higher education was only obtained by 57.8% of women in Kongo Central. Age at first intercourse was lower in Kongo Central (81.1% prior to age 18) versus Kinshasa (56.5% prior to age 18). Economic indicators were more optimal for women in Kinshasa versus Kongo Central, with higher proportions reporting paid work outside the household in the last seven days (56.6%_Kinshasa_ vs. 34.5%_KongoCentral_), having savings (33.3%_Kinshasa_ vs. 11.4%_KongoCentral_), and using a mobile money account (37.0%_Kinshasa_ vs. 13.3%_KongoCentral_). Within the relationship dyad, most women across provinces had a husband with less than tertiary education, and 20.2% and 33.5% of women in Kinshasa and Kongo Central, respectively, were married/cohabitating before age 18.

**Table 1:** Characteristics of partnered women interviewed for GBV module, by province, weighted

	Kinshasa (*n* = 707) n (%)^+^	Kongo Central (*n* = 692) n (%)^+^
Sociodemographic		
Household wealth		
Lowest	232 (33.1)	221 (32.1)
Middle	243 (34.6)	242 (35.1)
Highest	226 (32.3)	225 (32.8)
Marital Status		
Married	394 (56.2)	285 (41.4)
Living with partner	308 (43.8)	403 (58.6)
Age		
15–29	238 (33.9)	289 (42.0)
30–39	287 (40.8)	259 (37.6)
40–49	177 (25.3)	140 (20.4)
Education		
Primary or lower	62 (8.9)	290 (42.2)
Secondary or higher	639 (91.1)	398 (57.8)
Parity		
0	56 (8.0)	23 (3.3)
1–2	389 (55.5)	320 (46.6)
3 +	256 (36.5)	345 (50.1)
Age at first intercourse		
≦14	96 (13.8)	180 (26.7)
15—17	298 (42.7)	367 (54.5)
≧18	303 (43.5)	126 (18.8)
Economic		
Paid work outside the household, last 7 days	397 (56.6)	237 (34.5)
Savings (bank account, savings group, cash)	234 (33.3)	79 (11.4)
Use of mobile money account	259 (37.0)	91 (13.3)
Relationship Dyad		
Partner education		
Secondary or lower	385 (55.8)	622 (91.5)
Tertiary	304 (44.2)	58 (8.5)
Husband has other partners (yes or does not know)	42 (6.0)	81 (11.9)
Age at first marriage/cohabitation		
< 18	136 (20.2)	210 (33.5)
>= 18	537 (79.8)	417 (66.5)

In Kinshasa, over one in three women (35.3%) reported past-year IPV (physical, sexual, or emotional); in Kongo Central, prevalence was slightly lower at 29.7% (Table 2). Approximately one-fifth of women experienced past-year physical and/or sexual (i.e., contact) IPV (20.6%_Kinshasa_, 18.0%_KongoCentral_). The prevalence of physical IPV (12.5%_Kinshasa_, 11.9%_KongoCentral_) and sexual IPV (12.1%_Kinshasa_, 12.2%_KongoCentral_) was approximately equal across provinces, and about one quarter experienced emotional IPV (27.6%_Kinshasa_, 24.0%_KongoCentral_).

**Table 2:** Past-year IPV experience and help-seeking, by province, weighted

	Kinshasa (*n* = 703) n (%)^+^	Kongo Central (*n* = 684) n (%)^+^
IPV, past 12 months		
Any IPV (physical, sexual or emotional)	246 (35.3)	202 (29.7)
Contact IPV (physical or sexual)	144 (20.6)	123 (18.0)
Physical IPV^ϕ^	87 (12.5)	81 (11.9)
Sexual IPV^ϕ^	85 (12.1)	83 (12.2)
Emotional IPV^ϕ^	193 (27.6)	163 (24.0)
Help-seeking, among those with any IPV in past 12 months		
Did not seek any help	180 (73.1)	132 (62.3)
Sought help	66 (26.9)	80 (37.7)
Sought any formal help^ϕ^	2 (0.9)	4 (1.8)
Sought any informal help^ϕ^	64 (25.9)	79 (37.4)
Received useful or very useful informal help, among those who sought informal help	41 (64.3)	71 (89.8)
Number of types of informal help accessed (Mean (SD) Range)	0.4 (0.7) 0–4	0.8 (1.2) 0–5

^+^n’s and percents weighted

^ϕ^categories are not mutually exclusive

IPV = intimate partner violence

**Fig. 1:**
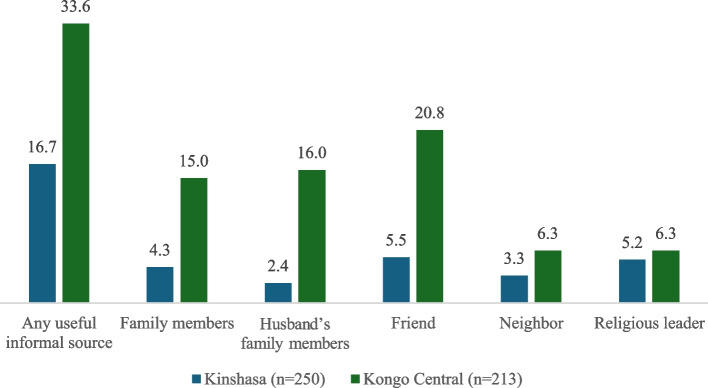
Weighted percentage reporting useful or very useful help from an informal source, among women who experienced IPV in the past 12 months, by source and province

Among those who experienced any past-year IPV, the majority did not seek help for their experiences (73.1% in Kinshasa and 62.3% in Kongo Central). Help-seeking among women who experienced IPV focused on informal sources (25.9%_Kinshasa_, 37.4%_Kongo Central_), such as from friends, neighbors, or family; on average, women sought help from less than one informal resource. Only two women in Kinshasa (0.9%) and three women in Kongo Central (1.8%) reported seeking help from a formal source (including police, women’s group, healthcare personnel, etc.). Among women who experienced IPV and sought help, 89.8% in Kongo Central and 64.3% in Kinshasa reported that they received useful or very useful help from informal services. The informal source most likely used and reported to be useful among women who experienced IPV was friends, with few women seeking and receiving useful help from neighbors and religious leaders in Kongo Central and neighbors and husband’s family in Kinshasa (Fig. 1).

**Fig. 2:**
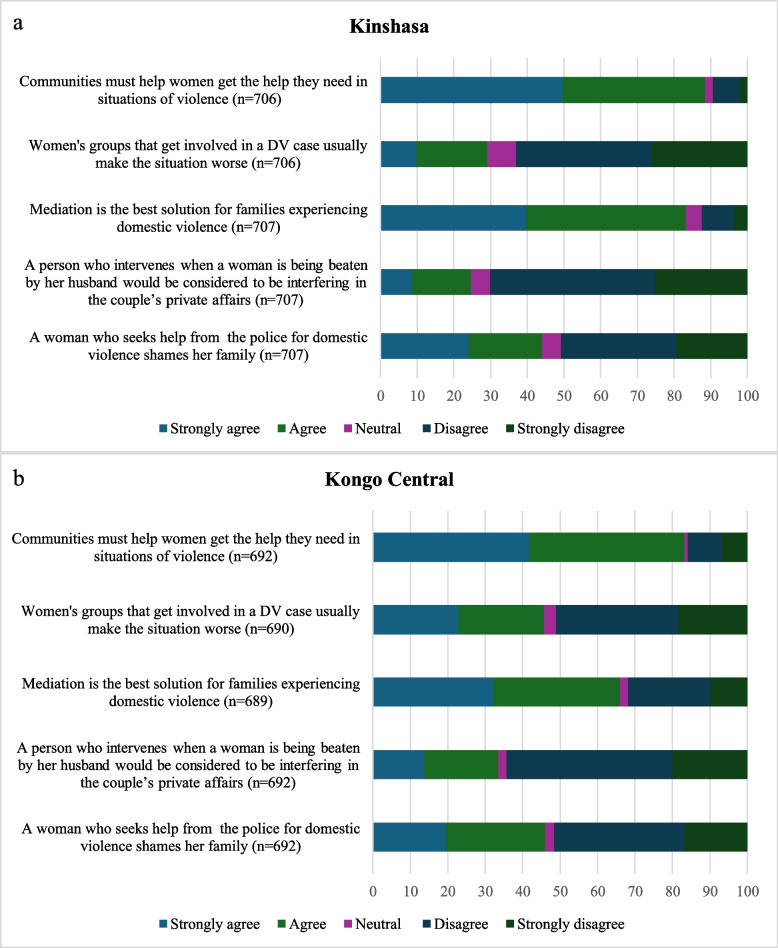
**a** Distribution of percent agreement with attitude items, Kinshasa, weighted. **b** Distribution of percent agreement with attitude items, Kongo Central, weighted

Patterns in attitudes towards IPV help-seeking were similar across provinces (Fig. 2). Around half or just over half of women disagreed that involving women’s groups could cause problems (63.1% disagree _Kinshasa_, 51.2% disagree _Kongo Central_) and that seeking help from the police brings shame (50.7% disagree _Kinshasa_, 51.6% disagree _Kongo Central_). Further, the majority disagreed with the statement that a person who intervenes in domestic violence is interfering with private affairs (70.2% disagree _Kinshasa_, 64.3% disagree _Kongo Central_). A small proportion disagreed with the statement that communities should help women if they need help with situations of violence (9.4% disagree _Kinshasa_, 15.9% disagree _Kongo Central_) and that mediation is the best solution for families experiencing domestic violence, though disagreement with mediation was much higher in Kongo Central (12.4% disagree _Kinshasa_, 31.8% disagree _Kongo Central_).

Bivariate associations between individual IPV help-seeking attitude items and individual-level help-seeking behaviors among women who experienced IPV were generally null (Table 3). However, in Kinshasa, women who experienced IPV and reported that involvement of women’s groups in domestic violence cases do *not* make things worse were significantly more likely (p-value < 0.001) to report having sought help for their experience of violence (OR: 2.57; 95% CI: 1.45, 4.49). A non-significant trend (p < 0.10) was observed between disagreeing that seeking help from police brings shame in Kongo Central (OR: 1.80; 95% CI: 0.96, 3.39) and disagreeing that intervening when a woman is beaten is inappropriate in Kinshasa (OR: 2.27; 95% CI: 0.93, 5.54) and help-seeking for IPV.

**Table 3:** Unadjusted associations of attitudes towards IPV response and informal and/or formal help-seeking, among women who experienced IPV, by province

	**Kinshasa**			**Kongo Central**		
	n (%)^+^	% sought help, by agree/disagree, weighted	OR^~^ (95% CI)	n (%)^+^	% sought help, by agree/disagree, weighted	OR^~^ (95% CI)
A woman who seeks help from the police for domestic violence shames her family						
Strongly agree, agree, or neutral	135 (52.2)	21.3	ref	93 (41.5)	29.8	ref
Disagree or strongly disagree	115 (47.8)	33.0	1.81 (0.79, 4.18)	120 (58.5)	43.3	1.80^ϕ^ (0.96, 3.39)
A person who intervenes when a woman is being beaten by her husband would be considered to be interfering in the couple’s private affairs						
Strongly agree, agree, or neutral	81 (29.0)	16.6	ref	63 (28.8)	38.8	ref
Disagree or strongly disagree	169 (71.0)	31.1	2.27^ϕ^ (0.93, 5.54)	150 (71.2)	37.2	0.94 (0.39, 2.25)
Mediation is the best solution for families experiencing domestic violence						
Strongly agree, agree, or neutral	222 (89.5)	28.1	ref	150 (73.1)	40.2	ref
Disagree or strongly disagree	28 (10.5)	16.4	0.50 (0.11, 2.35)	62 (26.9)	31.6	0.69 (0.31, 1.53)
Women's groups that get involved in a domestic violence case usually make the situation worse						
Strongly agree, agree, or neutral	119 (40.2)	16.6	ref	117 (55.0)	40.0	ref
Disagree or strongly disagree	131 (59.8)	33.8	2.57^***^ **(1.47, 4.49)**	96 (45.0)	34.8	0.80 (0.35, 1.84)
Communities must help women get the help they need in situations of violence						
Strongly agree, agree, or neutral	235 (92.4)	27.4	ref	190 (89.7)	38.1	ref
Disagree or strongly disagree	15 (7.7)	20.0	0.66 (0.18, 2.46)	23 (10.3)	33.6	0.82 (0.27, 2.53)

^~^Bivariate logistic regression accounting for complex survey design

^***^ *p* < 0.001, ^**^ *p* < 0.01, ^*^ *p* < 0.05, ^ϕ^*p* < 0.10; bolded values significant at *p* < 0.05

^+^n unweighted, percentage weighted

Pooled multivariable models between individual attitudes and help-seeking, adjusted for related demographic covariates are presented in Table 4. Disagreeing or strongly disagreeing, compared to strongly agreeing, agreeing or neutral, with attitude “a woman who seeks help from the police for domestic violence shames her family” was associated with increased odds of help-seeking (aOR:1.73; 95% CI: 1.03, 2.89). No other attitudes were significantly associated with help-seeking within multivariable models, though some covariates displayed associations with formal and/or informal help-seeking. Specifically, age > 39, as compared to age < 30, was associated with increased help-seeking, and residence in Kinshasa, compared to Kongo Central, was associated with reduced help-seeking in some models, but not in others.

**Table 4:** Multivariable associations between individual IPV attitude measures and help-seeking, among women who experienced IPV, Kinshasa and Kongo Central Provinces Pooled, weighted

	Formal and/or informal help-seeking
	aOR^~^ (95% CI)
A woman who seeks help from the police for domestic violence shames her family (*n* = 458)	
Strongly agree, agree, or neutral	ref
Disagree or strongly disagree	1.73^*^ (1.03, 2.89)
Wealth	
Lower	ref
Middle	0.64 (0.37, 1.10)
Highest	1.01 (0.59, 2.07)
Marital status	
Living w/partner	ref
Married	0.83 (0.47, 1.46)
Age	
< 30	ref
30–39	1.13 (0.70, 1.84)
> 39	1.65^+^ (0.96, 2.85)
Site	
Kongo Central	ref
Kinshasa	0.58 (0.30, 1.12)
A person who intervenes when a woman is being beaten by her husband would be considered to be interfering in the couple’s private affairs (*n* = 458)	
Strongly agree, agree, or neutral	ref
Disagree or strongly disagree	1.36 (0.72, 2.57)
Wealth	
Lower	ref
Middle	0.61^+^ (0.35, 1.05)
Highest	1.10 (0.60, 2.03)
Marital status	
Living w/partner	ref
Married	0.85 (0.47, 1.53)
Age	
< 30	ref
30–39	1.10 (0.66, 1.82)
> 39	1.62^+^ (0.93, 2.83)
Site	
Kongo Central	ref
Kinshasa	0.55^+^ (0.28, 1.05)
Mediation is the best solution for families experiencing domestic violence (*n* = 457)	
Strongly agree, agree, or neutral	ref
Disagree or strongly disagree	0.60 (0.29, 1.23)
Wealth	
Lower	ref
Middle	0.60^+^ (0.34,1.04)
Highest	1.09 (0.59, 2.01)
Marital status	
Living w/partner	ref
Married	0.88 (0.48, 1.58)
Age	
< 30	ref
30–39	1.21 (0.74, 1.98)
> 39	1.74^*^ (1.02, 2.97)
Site	
Kongo Central	ref
Kinshasa	0.49^*^ (0.25, 0.97)
Women's groups that get involved in a domestic violence case usually make the situation worse (*n* = 458)	
Strongly agree, agree, or neutral	ref
Disagree or strongly disagree	1.30 (0.75, 2.24)
Wealth	
Lower	ref
Middle	0.62^+^ (0.36, 1.06)
Highest	1.01 (0.60, 2.04)
Marital status	
Living w/partner	ref
Married	0.85 (0.47, 1.56)
Age	
< 30	ref
30–39	1.15 (0.71, 1.86)
> 39	1.65^+^ (0.95, 2.87)
Site	
Kongo Central	ref
Kinshasa	0.53^*^ (0.28, 0.98)
Communities must help women get the help they need in situations of violence (*n* = 458)	
Strongly agree, agree, or neutral	ref
Disagree or strongly disagree	0.82 (0.36, 1.86)
Wealth	
Lower	ref
Middle	0.63^+^ (0.37, 1.07)
Highest	1.12 (0.60, 1.08)
Marital status	
Living w/partner	ref
Married	0.85 (0.47, 1.53)
Age	
< 30	ref
30–39	1.14 (0.70, 1.87)
> 39	1.70^*^ (1.00, 2.90)
Site	
Kongo Central	ref
Kinshasa	0.55^+^ (0.28, 1.05)

^~^Adjusted odds ratio from multivariable logistic regression adjusting for site (Kinshasa/Kongo Central) and complex survey design; all variables specified within column were included in multivariable model, separately by attitude measure

^***^ *p* < 0.001, ^**^ *p* < 0.01, ^*^ *p* < 0.05, ^+^*p* < 0.10; bolded values significant at *p* < 0.05

## Discussion

Approximately one in five women in Kongo Central and Kinshasa experienced physical and/or sexual IPV within the past year, which is higher than many other global contexts [30]; this percentage increased to 35.3% in Kinshasa and 29.7% in Kongo Central when including emotional IPV. Results are in line with global research indicating low help-seeking and greater reliance on informal supports [5, 19, 42]. Specifically, the majority of physical, sexual, and emotional IPV survivors in both provinces did not seek any help for the violence they were experiencing (73.1%_Kinshasa;_ 62.3%_Kongo Central)_, and patterns indicated predominant reliance on informal supports (25.9%_Kinshasa_, 37.4%_Kongo Central_), such as friends and family members. Results are consistent with 2013 DHS estimates indicating that 30.7% and 24.4% of physical/sexual IPV survivors in Kinshasa and Kongo Central, respectively, sought help for their experiences [31], and indicate that despite attention to service provision, help-seeking has not increased within nearly ten years. Moreover, formal help-seeking was lower than prior research from 31 LMICs of 3% [5, 42], hovering around one percent in each province (0.9%_Kinshasa_; 1.8%_Kongo Central_).

Results surrounding help-seeking attitudes may help us understand barriers to care-seeking in this high violence context—specifically, attitudes supportive of the use of formal resources (police and women’s groups) were less prevalent, whereas they were more common for informal supports (communities, mediation, and people intervening). Notably, however, only disagreeing with the attitude “a woman who seeks help from the police for domestic violence shames her family” was associated with increased odds of help-seeking within multivariable models. Shifting attitudes surrounding formal structures may be key to increasing help-seeking—to increase use of formal services in DRC and similar high violence contexts, the normative environment that enables IPV and prevents help-seeking must shift to be more supportive of survivors’ goals. Simultaneously, informal sources, who are most often sought, should be equipped with helpful tools to respond to IPV.

Despite availability, women will only seek formal services if they feel the services will improve their situation and not escalate abuse and/or increase stigmatization [5, 28]. Substantial recent efforts have sought to improve both service availability, specifically within health facilities, and the normative context that normalizes violence and prevents women from seeking care [37]. Despite these efforts, across the two provinces, past-year help-seeking from various formal structures (doctor/medical professional, police, lawyer, social service organization, or a violence support program/hotline) was negligible (less than 2%). While the DRC National Roadmap of the Call to Action for Protection against Gender-Based Violence explicitly highlights IPV as a predominant form of GBV by definition [43], the legal context may not adequately respond to IPV survivors’ needs, including by not expressly prohibiting marital rape nor by criminalizing domestic violence specifically [44]. Against this backdrop, it is unsurprising that seeking help from police or lawyers was non-existent. While the legal context remains imperfect, linkage to medical service, social services, and violence support programs/hotlines is imperative to mitigate the health impacts of IPV [6, 9]. Particularly in cases of extreme physical and/or sexual violence, ensuring that providers are trained and implement trauma-informed care, such as the L.I.V.E.S curriculum, as recommended by the World Health Organization [45], is critical. Coupled with accessible services and trauma-informed provider trainings, demand generation programs, such as the Stop the Bus! campaign implemented in South Africa, Zimbabwe, and Ghana, which provided services via a mobile bus while simultaneously educating communities about GBV [46], can further help ease survivors’ concerns surrounding seeking formal services.

Importantly, IPV survivors reported seeking help from on average less than one informal support, meaning there was potentially just one opportunity for a bystander to help the survivor, by either minimizing blame or encouraging her to seek services. Further, not all informal help sought was seen as helpful—in Kinshasa, two-thirds (64.3%) of women who experienced IPV and sought help found that help was useful or very useful, though these proportions were higher in Kongo Central (89.8%). Helpfulness further varied by source and context (20.8% _Kongo Central_ vs. 5.5% _Kinshasa_), including by husband’s family members (16.0% _Kongo Central_ vs. 2.4% _Kinshasa_) and own family members (15.0% _Kongo Central_ vs. 4.3% _Kinshasa_). Previous research in the South Kivu providence of the DRC (Eastern DRC) describes that trust in community support systems, such as counseling by chiefs, mediation structures, and family elders, has deteriorated given ongoing conflict, high alcohol use, and weak leadership structures [47]. Helpfulness of informal help seems to be particularly of issue in the capital province (Kinshasa), potentially due to less stable community and familial relationships in urban areas. In conflict-ridden settings, including many parts of the DRC, working to restore community trust will be an important first step in counteracting harmful community norms. Programs such as Communities Care have proven valuable in increasing comfort in conversations surrounding IPV and equipping bystanders (i.e., informal supports) with messages of encouragement should they learn of IPV in their community [48–50]. This model is in line with the assessed attitude “communities must help women get the help they need in situation of violence”, where 88.6% of women in Kinshasa and 83.2% of women in Kongo Central, respectively, agreed or strongly agreed with this statement. Community-based models and programs, such as Communities Care, may further lift the onus from any one woman by providing strength in shared experiences and group accountability.

This research is not without limitations. Foremost, the PMA samples in the DRC are limited to two provinces, Kongo Central and Kinshasa, covering small geographic areas in the Western DRC with < 25% of the population; study findings are only generalizable at the provincial level and notably, these provinces report lower prevalence of IPV than those in the Eastern DRC [31]. The present analysis is also limited to women married or living together as if married, and excludes women who are divorced or separated. Moreover, level of agreement varied widely by IPV help-seeking attitude and in preliminary exploratory factor analyses, did not scale into a single latent construct. The items for help-seeking attitudes were based on preliminary research in Nepal [21], and indicate a further need for research in this area and particularly within the sub-Saharan African context. Further, given power limitations, multivariable models are pooled across provinces. Help-seeking data are also not disaggregated by subtype of IPV, nor can threshold or severity be discerned. While these data provide important insights into help-seeking attitudes within Kongo Central and Kinshasa provinces, qualitative research is needed to better understand the utility of available services and survivors’ barriers to help-seeking.

## Conclusions

These provincial level data are the only population-based estimates of IPV in DRC within the last ten years, which holds substantial implications for monitoring and evaluation research, though 2023 Demographic and Health Survey data are forthcoming. Recent investments into IPV prevention and response in the DRC, including related research, have been largely concentrated in conflict-affected areas in the Eastern DRC [36, 51], despite previous data indicating that IPV is an issue nationally. Continuous data collection at the national level is imperative to understand the types of violence women are affected by and potential demand for services, including those that may be limited by attitudes. There has been substantial recent IPV intervention innovation within the DRC [52, 53] and continued investment in population-based data can greatly assist in monitoring and evaluation of these programs, as well as monitoring the impact of national policies for IPV prevention, response, and justice.

## Data Availability

The datasets generated and/or analyzed during the current study are available in the Performance Monitoring for Action repository: https://www.pmadata.org/
